# Gene Expression Analysis of Non-Clinical Strain of *Aspergillus fumigatus* (LMB-35Aa): Does Biofilm Affect Virulence?

**DOI:** 10.3390/jof6040376

**Published:** 2020-12-18

**Authors:** Teresa D. Rebaza, Yvette Ludeña, Ilanit Samolski, Gretty K. Villena

**Affiliations:** Laboratorio de Micología y Biotecnología “Marcel Gutiérrez—Correa”, Universidad Nacional Agraria La Molina, 15026 Lima, Peru; damarisrebaza@gmail.com (T.D.R.); yludena@lamolina.edu.pe (Y.L.); isamolski@lamolina.edu.pe (I.S.)

**Keywords:** *Aspergillus fumigatus*, biofilms, gene expression, virulence

## Abstract

*Aspergillus fumigatus* LMB-35Aa, a saprophytic fungus, was used for cellulase production through biofilms cultures. Since biofilms usually favor virulence in clinical strains, the expression of the related genes of the LMB 35-Aa strain was analyzed by qPCR from the biomass of planktonic cultures and biofilms developed on polyester cloth and polystyrene microplates. For this, virulence-related genes reported for the clinical strain Af293 were searched in *A. fumigatus* LMB 35-Aa genome, and 15 genes were identified including those for the synthesis of cell wall components, hydrophobins, invasins, efflux transporters, mycotoxins and regulators. When compared with planktonic cultures at 37 °C, invasin gene *calA* was upregulated in both types of biofilm and efflux transporter genes *mdr4* and *atrF* were predominantly upregulated in biofilms on polystyrene, while *aspHs* and *ftmA* were upregulated only in biofilms formed on polyester. Regarding the transcription regulators, *laeA* was downregulated in biofilms, and *medA* did not show a significant change. The effect of temperature was also evaluated by comparing the biofilms grown on polyester at 37 vs. 28 °C. Non-significant changes at the expression level were found for most genes evaluated, except for *atrF*, *gliZ* and *medA,* which were significantly downregulated at 37 °C. According to these results, virulence appears to depend on the interaction of several factors in addition to biofilms and growth temperature.

## 1. Introduction

Filamentous fungi are widely distributed in nature due to their saprophytic condition and their capability to grow in several substrates and surfaces. Additionally, fungi are biotechnologically important due to their ability to produce enzymes, organic acids, and diverse secondary metabolites. Fungi of industrial use are generally recognized as safe and in this sense, it is important to assess the expression of the pathogenicity or virulence genes under different growth and culture conditions.

The *Aspergillus* group, mainly *A. niger*, is widely used in biotechnology [1]. *A. fumigatus* is gaining interest because of its plant polysaccharide modifying and degrading enzymes [2,3,4]. Particularly, the production of neutral and alkaline endoglucanases, which are in high demand for the textile biofinishing process [5], has been reported for this fungus [6]. However, *A. fumigatus* has also been widely recognized as an opportunistic pathogen which is responsible for many reactions from allergies to invasive pulmonary aspergillosis [7,8].

In clinical strains, certain genes have been reported as key factors for the virulence and pathogenicity of *A. fumigatus*, and their expression is also related to the formation of biofilms in affected tissues.

Although virulence may depend on the isolation origin of strains (environmental or clinical), there is not a specific group of genes in *A. fumigatus* that determines the virulence level. Virulence factors do not include essential genes for normal growth but are associated with some biological processes mainly involved in cell wall structure, thermotolerance, response to stress, signaling, toxin synthesis, nutrition and survival in the host [7,9,10,11].

On the other hand, biofilm formation is a natural form of growth in fungi, on both biotic and abiotic surfaces, which confer them survival advantages related to metabolic performance and stress resistance. Fungal biofilms have gained much industrial importance, however, at the same time, they can be considered as a virulence factor for opportunistic fungi. Differential gene expression occurs in biofilm as compared with planktonic growth. Some genes expressed in biofilms encode transcription and translation factors, regulators, and those involved in ribosomal protein synthesis and protein turnover, multi-drug resistance transporter genes, enzymes for extracellular matrix synthesis, extracellular enzymes as well as genes for adherence and secondary metabolism (toxins) [12,13].

In this research, *A. fumigatus* LMB-35Aa, an alkaline cellulase producing strain isolated from soil [14], was successfully cultured as a biofilm to improve their cellulase productivity, as has been previously reported for other *Aspergillus* species [15,16]. Nevertheless, considering the saprophytic condition of this strain and its genetic divergence from clinical isolates [17], it was also important to assess the expression of the main genes involved in the pathogenicity and virulence in biofilms. 

Multispecies fungal biofilms have great potential for cellulose conversion [18]. However, the industrial production of cellulases, as well as mixed and single species biofilms should be considered. In this study, the reason for using the single biofilms of *A. fumigatus* LMB-35Aa was related to our interest in producing neutral alkaline endoglucanases for use in the textile industry.

Complementarily and according to the systems biology approach, molecular tools are useful to assess bioprocess optimization in industrial biotechnology [19], but most scientific reports are focused on the transcriptomic analysis of clinical strains [7,20], so that information is still incipient for industrial biofilms [3].

## 2. Materials and Methods

### 2.1. Fungal Strain

*A. fumigatus* LMB-35Aa [14] was used throughout this study. The strain was maintained on potato dextrose agar (PDA). For inoculum preparation, before each experiment, the strain was grown in flasks with PDA during 72 h at 37 °C. Then, the spores were washed with 0.1% (*v*/*v*) Tween 80 solution and diluted until obtaining a concentration of 10^6^ spores/mL which was used as inoculum.

### 2.2. Culture Medium and Growth Conditions

Cellulase production medium was used as reported before [21], except that the carbon source was replaced by 0.5% *(w/v)* carboxymethyl cellulose (CMC).

For biofilms’ cultures on polyester support, flasks containing a pre-weighed 3.1 × 3.1 cm piece of cloth and 70 mL of distilled water were used. Each flask was inoculated with a 3% (*v*/*v*) of spore suspension and incubated with agitation (175 rpm) at 28 or 37 °C (according to the experiment) for 15 min to allow the attachment of spores. The unbound spores were washed twice with sterile distilled water in agitation at the same conditions. Finally, polyester cloths were transferred to 250 mL sterile flasks containing 70 mL of cellulase production medium. Inoculated flasks were incubated at 28/37 °C in a shaker bath at 175 rpm for 72 h.

For the biofilm cultures on polystyrene, flat-bottom 12-well polystyrene microtiter plates were used as a surface for biofilm formation [22]. Each well containing 3 mL of cellulase production medium was inoculated with 90 µL of spore suspension and then incubated without agitation at 37 °C for 15 min. After that, the medium was removed by pipetting and the plate wells were washed three times with distilled water. Finally, each well was refilled with 3 mL of cellulase production medium and microplates were incubated at 37 °C without agitation for 72 h.

For submerged culture, 250 mL flasks containing 70 mL of cellulase production medium were inoculated with 3% (*v*/*v*) of spore suspension and incubated at 28 or 37 °C for 72 h in a shaker bath at 175 rpm.

In all cases, for biomass harvesting, biofilms and free mycelium were washed three times with distilled water and maintained at −80 °C until RNA extraction.

### 2.3. Qualitative Endoglucanase Activity Assay

Qualitative assays of enzymatic activity were performed at different pH’s from 4.8 to 9.4 according Vega et al. (2012) with certain modifications [6]. Briefly, 100 µL of culture supernatants of *A. fumigatus* LMB-35Aa biofilms, developed on polyester cloth during 72 h in cellulase production medium, were incorporated into wells of 0.6 cm in diameter, equidistantly distributed in glass plates containing screening medium (1.5% agar and 0.3% CMC in the corresponding buffer). The plates were incubated at 50 °C for 24 h. After the incubation time, a 0.5% Congo Red solution was added on the medium for 15 min at room temperature and then washed with 1 M NaCl. Staining and washings were carried out in an orbital shaker at 50 rpm. The development of a clear zone (halo) around the wells was considered as a positive result of endoglucanase activity. Acetate buffer 50 mM (pH 4.8), borax buffer 50 mM (pH 7.6), and glycine buffer 50 mM (pH 8.4 and pH 9.4) were used correspondingly.

### 2.4. Quantitative Endoglucanase Activity

Quantitative assays of enzymatic activity were performed in 96-well microplates using 3,5-dinitrosalicylic acid (DNS) reagent according to the method described by Xiao et al. (2005) [23] with certain modifications. Briefly, a 30 μL aliquot of diluted culture supernatant was added into each microwell containing 30 μL of 1% CMC as a substrate prepared in 50 mM of the corresponding buffer (pH 4.8, 7.6, 8.4 or 9.4; see Section 2.3). After 30 min of incubation at 50 °C, 90 μL of DNS reagent was added into each well and incubated at 95 °C for 5 min. Following the color development, a 100 μL aliquot of each sample was transferred to a flat-bottom 96-well microplate and the absorbance was measured at 540 nm in a RT-2100C microplate reader (Rayto). An enzyme blank and substrate blank were also included in each assay. The concentration of glucose released by the secreted enzymes was determined by interpolating from a standard curve constructed with known concentrations of glucose. One enzyme unit (U) was defined as the amount of enzyme required to release 1 μmol of reducing sugar equivalents per minute under the defined assay conditions.

### 2.5. Extracellular Protein Determination

Soluble extracellular protein concentration was determined at 550 nm by Lowry’s colorimetric method using the Folin–Ciocalteu reagent with bovine serum albumin (BSA) as the protein standard [24].

### 2.6. Confocal Scanning Laser Microscopy (CSLM)

For the microscopy analysis, biofilm cultures on polyester cloth were developed as described above in Section 2.2. After 72 h of growth, the biofilms were washed three times with distilled water and then placed in 50 mM phosphate buffer (pH 7.4). Fluorescein isothiocyanate (FITC) and Concanavalin A (ConA) were used to stain the fungal hyphae and extracellular matrices, respectively. For that, stocks solutions of each dye were prepared in 10 mM phosphate buffer (pH 7.4) and mixed to a final concentration of 10 µg/mL (FITC) and 50 µg/mL (ConA). Biofilms were stained with 15 µL of this mix for 30 min in the dark at room temperature [25]. Finally, the biofilms were washed with 10 mM phosphate buffer four times and a FLUOVIEW FV1200 confocal scanning laser microscope (Olympus Life Science) was used for the image analysis and acquisition. FITC was excited/monitored at 458/488 nm and ConA was excited/monitored at 490/515 nm.

### 2.7. RNA Extraction and cDNA Synthesis

Total RNA isolation was carried out using RNA Miniprep kit (Zymo Research^®^, Irvine, CA, USA) after grinding the biomass with liquid nitrogen. The quality and quantity of all RNA samples were analyzed in a NanoDropTM 2000c spectrophotometer (Thermo Scientific^®^, Waltham, MA, USA) and by agarose gel electrophoresis. In all cases, cDNA was synthesized from 2 µg of total RNA in a 25 µL final volume using a reverse transcription mix containing 200 U of M-MLV RT (Promega^®^, Madison, WI, USA)), 0.5 mM dNTP mix, 0.5 μg of Oligo(dT)15 and 25 U of RNAse inhibitor. Reaction tubes were incubated at 42 °C for 60 min and stored at −20 °C until required for qPCR analysis.

### 2.8. Identification of Virulence Genes in A. fumigatus LMB-35Aa Genome

*A. fumigatus* Af293 clinical strain was used as a reference [26] for the screening of virulence genes in *A. fumigatus* LMB-35Aa genome, which included those encoding synthesis of cell wall components, hydrophobins, invasins, efflux transporters, mycotoxins and regulators.

The selection of virulence genes from the clinical strain *A. fumigatus* Af293 and nucleic sequence alignment was done using BLAST (Basic Local Alignment and Search Tool) to find the corresponding genes in *A. fumigatus* LMB-35Aa genome (Accession PRJNA298653) [14] with a sequence similarity higher than 98%. Table S1 indicates the location of the corresponding genes in the *A. fumigatus* LMB-35Aa genome, including the scaffold number and length (bp).

### 2.9. Primers Design

Primer Quest SM software (Integrated DNA Technologies, Inc.) was used to the design primers from the *A. fumigatus* LMB-35Aa genome sequence available in GenBank (PRJNA298653). The primers used in this study are listed in Table S2.

### 2.10. Gene Expression Analysis by qPCR

Gene expression was analyzed by quantitative real-time polymerase chain reaction (qPCR) with KAPA SYBR Fast kit (KAPA Biosystems^®^, Wilmington, MA, USA) according to the manufacturer’s protocol. qPCR was performed in a CFX96 Real Time PCR Detection System (Bio-Rad^®^, Hercules, CA, USA). Each reaction well with a final volume of 10 μL contained 1 μL of cDNA template and 0.3 μM of each forward and reverse primer (10 mM). The amplification process included an activation step at 95 °C for 3 min followed by 40 cycles at 95 °C for 3 s and 60 °C for 20 s (annealing and extension). After that, a melting curve analysis was included at 60–95 °C to confirm the specific amplification, according to the melting temperature (Tm) expected for each amplicon. Each reaction was carried out in triplicate and each plate included non-target controls. Two independent biological replicates were analyzed. After amplification, the cycle threshold (Ct) number was recorded for the reference and target genes. The amplification efficiency of each pair of primers was validated experimentally from the slope of the log-linear range of the calibration curve constructed with the serial dilutions of target cDNA.

The relative gene expression was calculated according to Hellemans et al. (2007) [27], which constitutes a modified Delta-Delta Ct method by considering the amplification efficiency of target genes and multiple reference genes for the improved normalization of relative quantities. ẞ-Tubulin (*btub*) (F: 5′-TTCACTGCTATGTTCCGTCG-3′; R: 5′-TCGTTCATGTTGCTCTCGG-3′) [28] and elongation factor (*tef1*) (F:5′CCATGTGTGTCGAGTCCTTC-3′, R:5′-GAACGTACAGCAACAGTCTGG-3′) were used as reference genes.

## 3. Results

### 3.1. Influence of pH and Temperature on A. fumigatus LMB-35Aa Endoglucanase Activity of Biofilms Formed on Polyester

**Figure 1:**
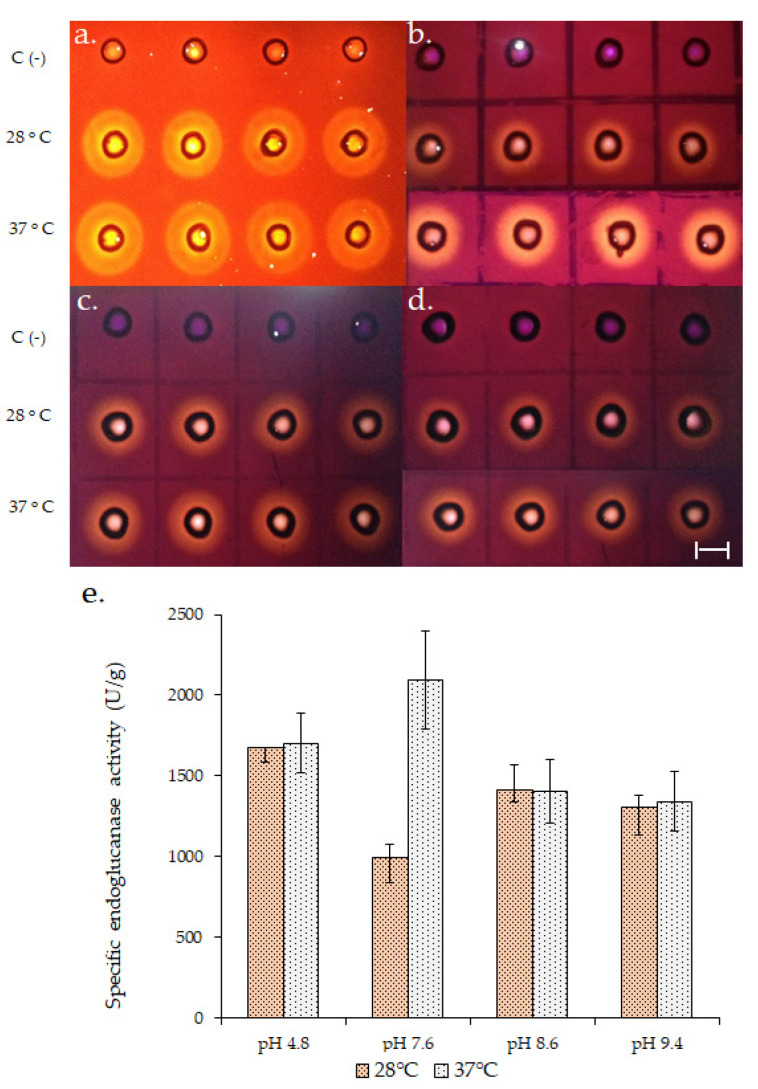
Endoglucanase activity (EG) of *A. fumigatus* LMB-35Aa biofilms developed on polyester cloth at 72 h of growth and 28 or 37 °C. The upper panel shows a qualitative assay for EG at (**a**) pH 4.8; (**b**) 7.6; (**c**) 8.6 and (**d**) 9.4. C (-) represents the negative control for the assay. Specific EG activity (**e**) was calculated by quantitative assay. Specific activity = EG (U/L)/soluble secreted protein (g/L). Scale bar represents 1 cm.

Endoglucanase production was compared at 28 and 37 °C until 120 h of growth and maximum enzymatic title was obtained at 72 h at both temperatures (Teresa D. Rebaza and Gretty K. Villena. Universidad Nacional Agraria La Molina, Lima, Perú. Diploma Thesis, 2019). At this point, the qualitative and quantitative endoglucanase activity (Figure 1) of the biofilms grown at 37 °C was higher at pH 7.6. At least a two-fold increase in the specific activity (units of enzyme/g of secreted protein) was obtained at 37 °C at pH 7.6 so that this temperature could be used for subsequent assays.

### 3.2. Influence of Biofilm Formation on A. fumigatus LMB-35Aa Virulence-Related Gene Expression

As better conditions for endoglucanase production and activity, 37 °C and pH 7.6, respectively, could also be favorable for pathogenesis and virulence, a gene expression analysis was performed to evaluate if, in addition, biofilm formation affects the expression of virulence-related genes. For that, 15 virulence-related genes described for the clinical strain *A. fumigatus* Af293, including genes for the synthesis of cell wall components, hydrophobins, invasins, efflux transporters, mycotoxins and regulators, were selected after searching for them in the *A. fumigatus* LMB-35Aa genome. Virulence-related genes found in the genome are described in Table 1.

**Figure 3:**
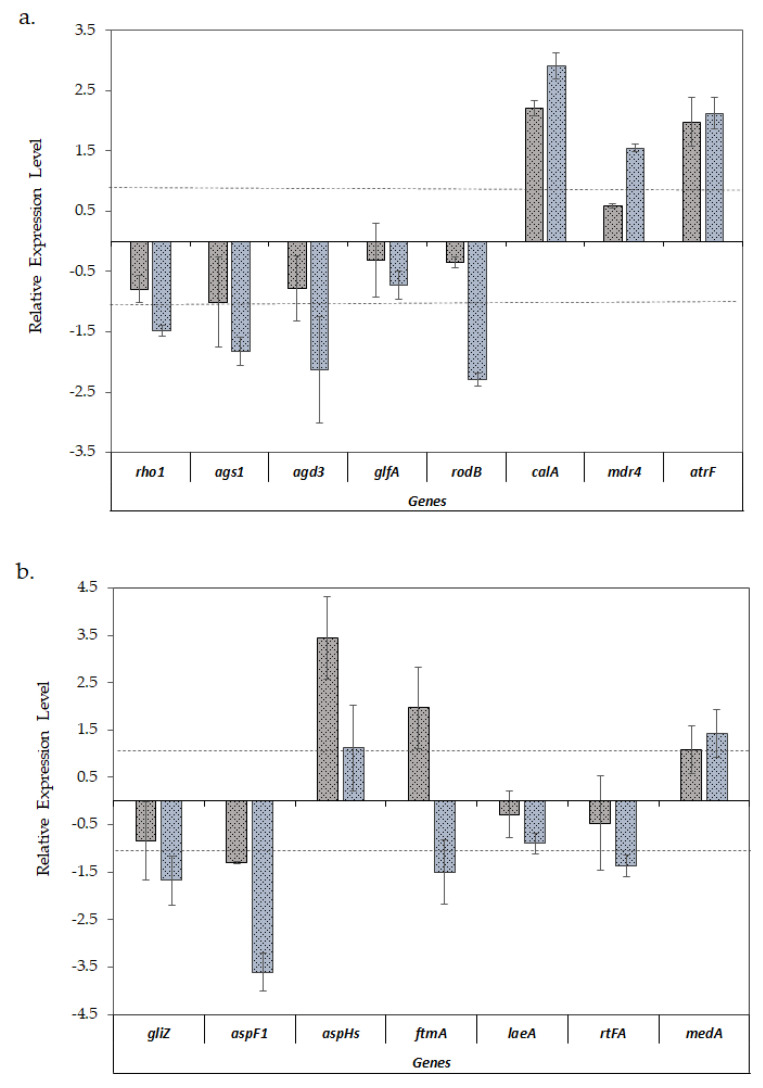
Relative expression ±S.D. of the virulence-related genes of A. fumigatus LMB-35Aa between the biofilms developed on polyester (grey bars) and polystyrene (blue bars) vs. planktonic cultures: (**a**) analysis of genes of the cell wall structure, hydrophobin, invasin and efflux transporters; and (**b**) the genes involved in mycotoxin biosynthesis, adhesion, and secondary metabolism. Gene expression levels were normalized using β-tub and tef1 as reference genes. The relative expression level is represented as a log_2_ fold change; dotted lines indicates log_2_ fold change thresholds of −1 and 1.

When comparing the expression of virulence-related genes (Figure 3a,b), a similar expression pattern was observed in both biofilm models with the exception of fumitremorgin biosynthesis *ftmA* gene, which was upregulated in the biofilms formed on the polyester cloth and downregulated in the biofilms formed on polystyrene, with respect to the planktonic cultures.

On the other hand, a differential gene expression was observed when the biofilms were compared with planktonic cultures. According to Figure 3a, all the genes involved in the cell wall structure (*rho1*, *ags1*, *agd3* and *glfA*) showed a lower expression level in biofilms, a pattern which was especially significant in the case of biofilms developed on polystyrene. This pattern was also observed in the case of the hydrophobin *rodB* gene. Conversely, invasin gene *calA* was significantly upregulated in both types of biofilms. With respect to ABC efflux transporters, genes *mdr4* and *atrF* were predominantly upregulated in biofilm formed on polystyrene.

In the opposite way, Figure 3b shows that the *aspHs* gene, which encodes for hemolysin, and *ftmA*, involved in mycotoxin biosynthesis, were upregulated only in biofilms on polyester, while *gliZ* and *aspF1* genes, encoding proteins involved in secondary metabolism, exhibited a significant downregulation in biofilms formed on polystyrene.

With respect to *medA*, involved in cell adhesion and biofilm formation, it had a slightly higher level of expression in the biofilms formed on polystyrene as compared with those formed on polyester, although in both cases these were not significant. Finally, with regard to regulator genes, *rtfA* was downregulated and the *laeA* gene, encoding for a secondary metabolism regulator, did not show any significant change when *A. fumigatus* formed biofilms.

### 3.3. Effect of Temperature on A. fumigatus LMB-35Aa Virulence-Related Genes Expression

Given that polyester supports are more suitable for fungal biofilm formation at an industrial level [13] and considering that a greater number of virulence-related genes were upregulated in the biofilms developed on this support, an additional experiment was carried out to evaluate if the temperature could affect the observed gene expression patterns.

**Figure 4:**
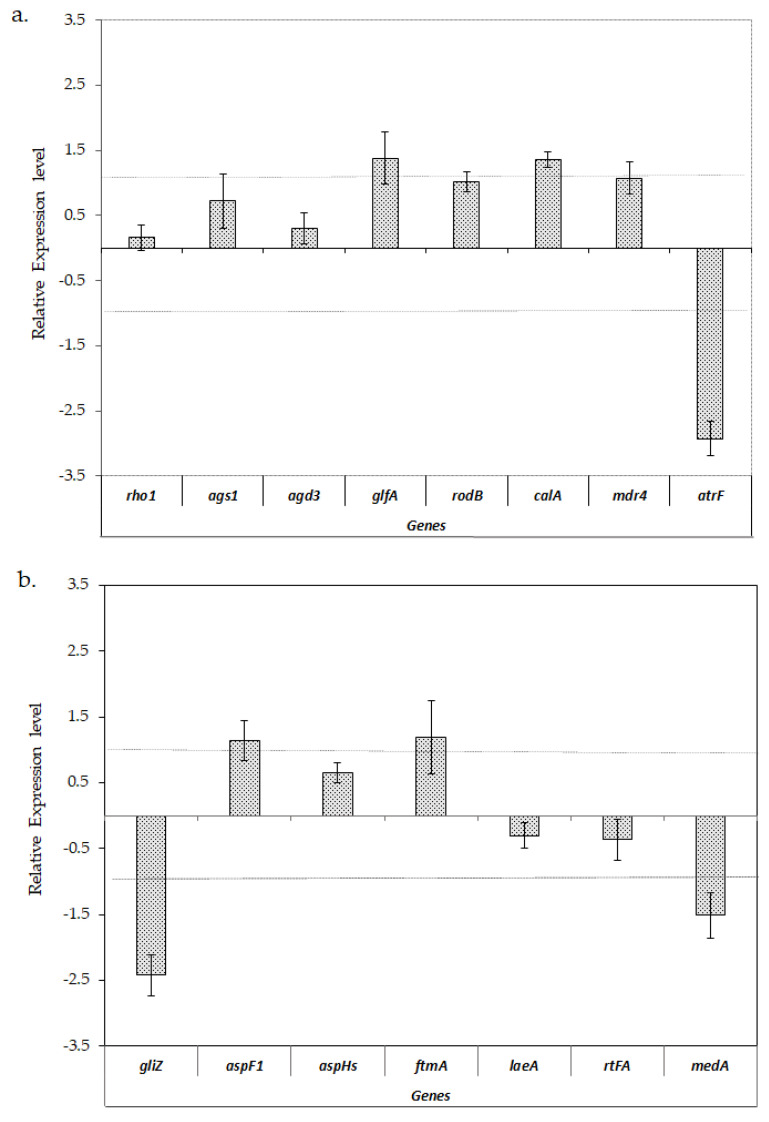
Relative expression ±S.D. of the virulence-related genes of *A. fumigatus* LMB-35Aa biofilms formed on polyester at 37 vs. 28 °C: (**a**) analysis of genes of the cell wall structure, hydrophobin, invasin and efflux transporters; and (**b**) the genes involved in mycotoxin biosynthesis, adhesion and secondary metabolism. Gene expression levels were normalized using *β-tub* and *tef1* as the reference genes. The relative expression level is represented as a log_2_ fold change; dotted lines indicates log_2_ fold change thresholds of −1 and 1.

Figure 4 shows that the expression levels of analyzed genes involved in cell wall structure did not show any significant change when the biofilms grew at 28 or 37 °C, with the exception of *glfA* and *calA,* which showed a slightly higher level of expression at 37 °C. With regard to the analyzed efflux transporters genes, *atrF* was significantly downregulated at 37 °C compared to 28 °C, while *mdr4* did not show any significant change in any condition. Most of the analyzed genes involved in mycotoxins biosynthesis and regulation did not show notable changes in their expression levels when the biofilms were formed at 28 or 37 °C, except for the *gliZ* gene, involved in gliotoxin biosynthesis, which was significantly downregulated at 37 °C.

## 4. Discussion

*A. fumigatus* is gaining great interest as an enzymes producer for biotechnological applications including lignocellulose conversion to added value products [2,4,49,50,51,52]. In this case, strain LMB-35Aa was selected as a neutral alkaline endoglucanase producer and grown as biofilms on polyester cloth in order to improve its enzymatic productivity.

Despite its saprophytic condition and not even being grouped with clinical strains, the expression of genes related to virulence in biofilms of *A fumigatus* LMB-35Aa were analyzed. Some particular genetic characteristics of this strain, related to secondary metabolism (SM) cluster variants include the lack of a 54 kb region (five genes from telomere-proximal fumigaclavine C cluster) in the chromosome 2, and a large inversion in the SM gene cluster 14 that contains a transcription factor, an oxidoreductase, and a hypothetical protein [17].

For *A. fumigatus*, biofilm formation is one of the most determining virulence factors [53,54]. In that sense, the strain LMB-35Aa has a good capacity to adhere to abiotic surfaces and form biofilms on polyester and polystyrene supports with a typical structure including an extracellular matrix, as it was also reported for different *Aspergillus* biofilms [13]. Several fungal constituents may be involved in the formation of biofilm in host cells. Associated with this, biofilms formed by the strain LMB-35Aa could be expected to express virulence-related genes, including cell wall components, invasins, and efflux transporters, among others [55].

The cell wall is mainly composed of polysaccharides like β-glucans and galactomannans and some genes like *rho1*, *ags1*, *agd3* and *glfA*, which are involved in the biosynthesis of β-glucans, α-glucans, galactosaminogalactans and galactomannans, respectively, which can also have an impact on virulence, increasing resistance to antifungals and concentrating the extracellular enzymes produced during growth, which are necessary aspects for colonization and tissue infection. Moreover, *rho1* might be required for *A. fumigatus* pathogenicity and internalization into lung epithelial host cells [29]. Gene *ags1* contributes significantly with at least 50% of the cell wall α-1,3-glucan content, however, it is not a determinant for virulence [56], while *agd3* encodes for a deacetylase of galactosaminogalactan, which is an important virulence factor, and induces adherence and biofilms formation [33]. In addition, a ∆*glfA* mutant of *A. fumigatus* produced slower growth and attenuated virulence since a diminished content of glucofuranose causes the thinning of the cell wall and an increased susceptibility to drugs [34]. Interestingly, none of these genes were upregulated in biofilms of LMB-35Aa, probably due to the time of growth (72 h) being much later than the first stages of biofilm formation. Probably, for the same reason, the *medA* gene, involved in biofilm formation and adherence [47,48], was slightly expressed.

By the other hand, gene *rodB*, encoding a Class I hydrophobin in *A. fumigatus*, was downregulated in biofilms, contrarily to the high expression found in a cellophane biofilm model developed by Valsecchi et al. (2018), although, at the same time, they found that the corresponding protein RodB analyzed by Western blots was present in the conidium cell wall but not in the hyphae of planktonic or biofilm cultures [36]. This means that the high gene expression does not always correlate with high protein production. Here, we found that the *rodB* gene was upregulated in our planktonic cultures (pellets) since sporulation occurred early inside the pellet.

Another important virulence gene, *calA*, was upregulated in both biofilm models, especially in biofilms on polystyrene. CalA is dispensable for adherence, but it is important as invasin and induces the host cell endocytosis of pathogenic *A. fumigatus* [37].

Drug efflux transporter genes *cmdr4* and *atrF*, which contribute to itraconazole resistance [57] and are induced by the presence of that drug [58], were also upregulated in biofilms. Even this being a typical resistance for clinical strains, it has also been reported as an environmental route of resistance development [59]. Most of the genes involved in mycotoxin biosynthesis were downregulated in biofilms, except *ftmA,* for the biofilms grown on polyester. In the same way, *aspHs*, a virulence factor which encodes for hemolysin, was slightly overexpressed. This gene has been proposed as a specific target for *A. fumigatus* detection by qPCR for in vivo infections [60]. Thus, it could be related to biofilm formation.

It was remarked that transcription factors are important for fungal pathogenicity because of their role in regulating the transcription of virulence-related pathways [61]. In this case, *medA* was poorly expressed. Additionally, *laeA* was downregulated and correlated with *gliZ* downregulation since LaeA is a transcriptional regulator of secondary metabolite gene clusters including gliotoxin [46].

Gene expression could be also influenced by growth temperature as reported by Sueiro-Olivares et al. (2015) [62] when analyzing the transcriptomes of *A. fumigatus* during the early steps of conidia germination after being grown at 24 and 37 °C. Between the 1249 differentially expressed genes, *gliZ* was upregulated at 37 °C.

Unexpectedly, in our case, when comparing the gene expression patterns of biofilms grown at 37 vs. 28 °C, only a slight but not significant expression of virulence-related genes was observed at 37 °C. However, the fact that some virulence factors such as *gliZ*, *atrF* and *medA* were repressed at this temperature is even more relevant.

This highlights that virulence is a multifactorial condition with many determinants acting together. For industrial purposes, even when *A. fumigatus* biofilm formation slightly induces the level of expression of virulence-related genes, it is not enough to attribute to it a leading role in the virulence for non-clinical strains. Perhaps, together with the influence of the temperature of growth, another signaling mechanism related with host interaction could be explored to discard the potential pathogenicity of the strain LMB-35Aa during biofilm formation.

## 5. Conclusions

Biofilm formation in the non-clinical *A. fumigatus* LMB-35Aa strain could lightly induce the upregulation of some virulence-related genes, including *calA*, *mdr4*, *atrF*, *aspHs* and *ftmA,* but also the influence of temperature was evident, especially for the downregulation of *gliZ*, *atrF* and *medA* at 37 °C. Further research using an in vivo biofilm model could contribute to a better understanding of the complex virulence phenomena.

## Figures and Tables

**Figure 2:**
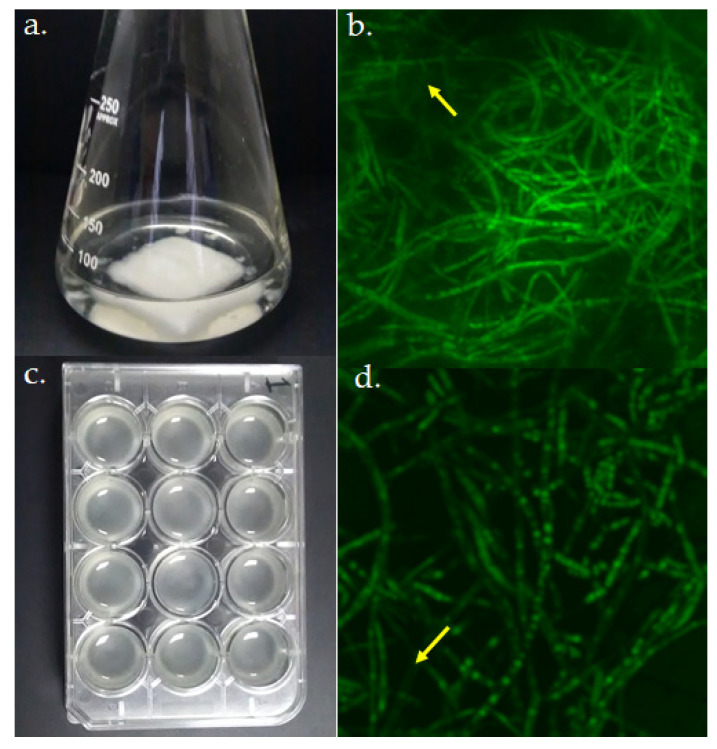
Biofilm models of *A. fumigatus* LMB-35Aa grown on (**a**) polyester and (**c**) polystyrene. Biofilm structure was analyzed by CSLM at 72 h of growth. Average projections of stained biofilms 40× images on (**b**) polyester and (**d**) polystyrene are shown. Green fluorescence depicts fungal viable cells; arrows indicate regions with the extracellular matrices of biofilms.

**Table 1:** Selected virulence-related genes found in the genome of the saprophytic strain LMB-35Aa, considering the clinical strain *A. fumigatus* Af293 as a reference.

Gene	Gene Function	Role Associated with Virulence	Reference
*rho1*	β-(1,3) glucan biosynthesis regulation	Regulation of cell wall composition and oxidative alkaline stress	[29]
*ags1*	α-(1-3) glucan biosynthesis	Conidia adhesion capacity and survival; late phagocytosis	[30,31]
*agd3*	Deacetylation of galactosaminogalactan (GAG)	Induces biofilms formation	[32,33]
*glfA*	Galactofuranose biosynthesis	Conidia germination and growth inside macrophages; resistance to antifungal drugs	[34,35]
*rodB*	Hydrophobin	Upregulation in biofilm conditions and in vivo	[36]
*calA*	Invasin	Invasion of epithelial and endothelial host cells through endocytosis induction	[37]
*mdr4*	ABC multidrug transporter	Azole resistance (clinical isolates)	[38]
*atrF*	ABC multidrug transporter	Azole resistance (environmental isolates)	[39]
*gliZ*	Gliotoxin biosynthesis regulation	Induces apoptosis and cytotoxicity	[40]
*aspf1*	Ribotoxin	Cytotoxicity, cell surface allergen	[41]
*aspHs*	Hemolysin biosynthesis	Hemolysis and cytotoxicity	[42]
*ftmA*	Fumitremorgins biosynthesis (tremorgenic toxins)	Cytotoxicity	[43]
*laeA*	Secondary metabolism master regulation	Induces gliotoxin and other secondary metabolites production and cytotoxicity of host cells	[44,45]
*rtfA*	Developmental and secondary metabolism regulation	Oxidative stress response, protease activity, adhesion capacity	[46]
*medA*	Developmental regulation	Regulates conidiogenesis, adherence to host cells and biofilm formation, damage of epithelial cells and stimulation of cytokine production	[47,48]

For the gene expression analysis, two biofilm models (Figure 2a,c) and two growth temperatures were evaluated and compared with planktonic cultures. Biofilms were morphologically evaluated by CLSM and in both cases, a typical mycelium organization and extracellular matrix were observed (Figure 2b,d).

## Supplementary Materials

The following are available online at https://www.mdpi.com/2309-608X/6/4/376/s1, Table S1: Virulence-related genes identified in *A. fumigatus* LMB-35Aa genome, considering the clinical strain *A. fumigatus* Af293 as reference, Table S2: Primer sequences for gene expression analysis through qPCR for main virulence-related genes found in *A. fumigatus* LMB-35Aa genome.
