# Supplementary material for: Gene Expression Analysis of Non-Clinical Strain of Aspergillus fumigatus (LMB-35Aa): Does Biofilm Affect Virulence?
Source: J Fungi (Basel). 2020 Dec 18;6(4):376. doi: 10.3390/jof6040376 (PMC7766361; doi:10.3390/jof6040376)
Supplement: Supplementary file 1 [file jof-06-00376-s001.pdf]

Table S1: Virulence related genes identified in *A. fumigatus* LMB35Aa genome, considering the clinical strain *A. fumigatus* Af293 as reference.

Gene ID	Gene name	Gene location	Similarity with *A. fumigatus* Af293 strain
AFUA_6G06900 (ID 3508842)	*rho1*	scaffold15_size1369881	99%
AFUA_3G00910 (ID 3506018)	*ags1*	scaffold5_size439689	99%
AFUA_3G07870 (ID: 3512474)	*agd3*	scaffold2_size2524342	99%
AFUA_3G12690 (ID: 3512175)	*glfA*	scaffold30_size544622	99%
AFUA_1G17250 (ID: 3510125)	*robB*	scaffold22_size414029	99%
AFUA_3G09690 (ID: 3511970)	*calA*	scaffold24_size814358	99%
AFUA_1G12690 (ID: 3510389)	*mdr4*	scaffold2_size2524342	99%
AFUA_6G04360 (ID: 3505316)	*atrF*	scaffold26_size551250	99%
AFUA_6G09630 (ID: 3508157)	*gliZ*	scaffold15_size1369881	98%
AFUA_5G02330 (ID:3505541)	*aspF1*	scaffold6_size935784	99%
AFUA_3G00590 (ID: 3505795)	*aspHs*	scaffold31_size151726	99%
AFUA_8G00170 (ID: 3504405)	*ftmA*	scaffold42_size84196	99%
AFUA_1G14660 (ID: 3509858)	*laeA*	scaffold53_size69050	99%
AFUA_2G01900 (ID: 3506941)	*rtfA*	scaffold13_size1331972	99%
AFUA_2G13260 (ID: 3513134)	*medA*	scaffold3_size965284	99%

Table S2: Primer sequences for gene expression analysis through qPCR for main virulence related genes found in *A. fumigatus* LMB-35Aa genome.

Gene name	Primers sequences (5'- 3')	Amplicon size (bp)
rho1	**F:** GATCTCCGAGGTTCTTCACTTC<br>**R:** GCAGCTCCTCAATGGTCTT	98
ags1	**F:** CCAACACCTGGAAGATGACC<br>**R:** AACACCGACCGATAGAAGGA	139
agd3	**F:** CGAGCAACACCAAGGAGAT<br>**R:** GGTGGCAAAGCCAATGAAG	125
glfA	**F:** GCTTCGGTGAGAAGGGTAAG<br>**R:** GCTTCTTGTAGCCGATGGT	90
rodB	**F:** CCAGAACAAGTGCGGTGA<br>**R:** TCAGGAGGCCGTAGTTGA	92
calA	**F:** TCCGGTGACACCATCTACT<br>**R:** GACGGTGAAGCCGAACTT	81
mdr4	**F:** CGTTCGGCAGAGGATGAA<br>**R:** CTCTGGGCGATGTGGATAAG	111
atrF	**F:** CAGCCCTCGTCGATGTTAAT<br>**R:** GCCAACTGGACCGAAATAGA	87
gliZ	**F:** AGCCTAACCGCTCGAGATA<br>**R:** CAGTGGGAACAGTGGTAGAAG	98
aspF1	**F:** TACCCGCACTGGTTCACTAA<br>**R:** GACGGTCACAGTCGGCTT	94
aspHs	**F:** AGTCCACTGGGACTGTCCAT<br>**R:** GCACCACCATACTTGTTCCA	108
ftmA	**F:** CCACGATGGCCCTATGTTT<br>**R:** GGATGTGCCGTAGAGGAATG	131
laeA	**F:** GTCTCGGACGGACTGATCTA<br>**R:** CGACGAAGGCTTCTGGATAC	118
rtfA	**F:** CTGAGGCCATTGAGGCATAC<br>**R:** CTTCACCAGCAGCCATATCC	87
medA	**F:** CGAAACATTGAAAAGGATGTG<br>**R:** CTGAGGGAAATCTGAAGGAAG	107
